# Non-invasive Transcranial Electrical Stimulation in Movement Disorders

**DOI:** 10.3389/fnins.2020.00522

**Published:** 2020-06-05

**Authors:** Jacky Ganguly, Aditya Murgai, Soumya Sharma, Dorian Aur, Mandar Jog

**Affiliations:** Movement Disorder Centre, London Health Sciences Centre, The University of Western Ontario, London, ON, Canada

**Keywords:** non-invasive brain stimulation (NIBS), transcranial electrical stimulation (tES), transcranial direct current stimulation (tDCS), transcranial alternating current stimulation (tACS), transcranial pulsed current stimulation (tPCS), transcranial random noise stimulation (tRNS)

## Abstract

Dysfunction within large-scale brain networks as the basis for movement disorders is an accepted hypothesis. The treatment options for restoring network function are limited. Non-invasive brain stimulation techniques such as repetitive transcranial magnetic stimulation are now being studied to modify the network. Transcranial electrical stimulation (tES) is also a portable, cost-effective, and non-invasive way of network modulation. Transcranial direct current stimulation and transcranial alternating current stimulation have been studied in Parkinson’s disease, dystonia, tremor, and ataxia. Transcranial pulsed current stimulation and transcranial random noise stimulation are not yet studied enough. The literature in the use of these techniques is intriguing, yet many unanswered questions remain. In this review, we highlight the studies using these four potential tES techniques and their electrophysiological basis and consider the therapeutic implication in the field of movement disorders. The objectives are to consolidate the current literature, demonstrate that these methods are feasible, and encourage the application of such techniques in the near future.

## Introduction

In movement disorders, non-invasive brain stimulation (NIBS) is an evolving therapeutic strategy. There is emerging evidence of network-level dysfunction in many neurological disorders. Movement disorders such as Parkinson’s disease (PD) (de Schipper et al., 2018), dystonia (Schirinzi et al., 2018), tremor (Benito-León et al., 2015), and ataxia (Falcon et al., 2016; Wu et al., 2018) may fit very well within this construct of network dysfunction to explain the pathophysiology and phenotypes. This paradigm shift of suggesting that the movement disorders are a result of dysfunction in multilevel, interconnected complex cortico-subcortical network rather than only being restricted to the basal ganglia has opened the possibility of modifying that network non-invasively by delivering electromagnetic stimulation. In addition, such an approach extends the neurophysiological substrate of movement disorders beyond chemical dysfunction or intracellular mechanisms. The concept that transcranial stimulation modifies surface and deep brain electrical networks is not intuitive due to the obvious question of penetration of such currents through the scalp and bone. However, interestingly, the current literature is suggesting that NIBS can modulate the complexity of the neural network and alter neural excitability potentially in cortical and deep brain structures. Since movement disorders involve structures at all these levels to potentially generate a disease phenotype, the application of NIBS to these conditions may be of particular interest. This intriguing new technology can not only help us to understand the pathophysiology of the movement disorders with a newer outlook but also be a new armamentarium in our therapeutic strategy for these disorders.

**FIGURE 1:**
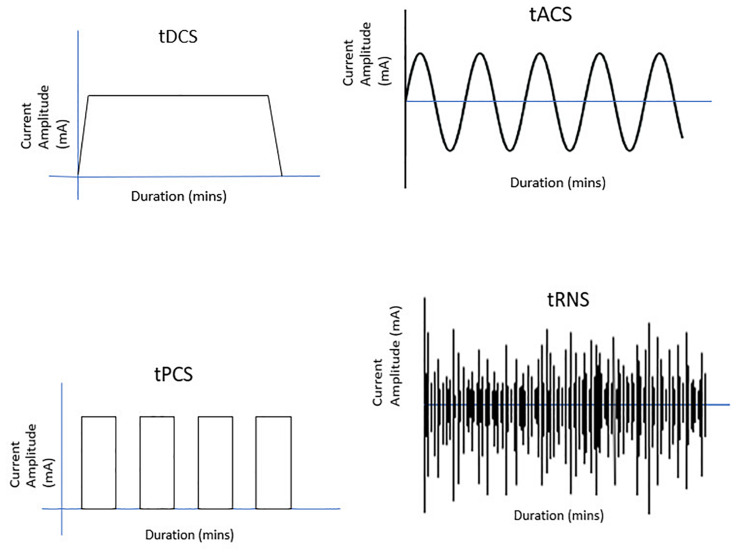
Different modes of transcranial electrical stimulation.

Repetitive transcranial magnetic stimulation (rTMS) has been studied most extensively in this regard. It has been evaluated in PD, dystonia, essential tremor, Huntington’s chorea, and chronic tic disorders like Tourette syndrome (Latorre et al., 2019). Four additional methods of NIBS, with transcranial electrical stimulation (tES), are also being evaluated as potential therapeutic options in neurodegenerative disorders—transcranial direct current stimulation (tDCS), transcranial alternating current stimulation (tACS), transcranial pulsed current stimulation (tPCS), and transcranial random noise stimulation (tRNS). The technique of tES involves the delivery of current to an individual’s scalp usually *via* two sponge electrodes. The current penetrates the scalp and is conducted to the brain area of interest, where it can alter neuronal excitability. In tDCS, a constant direct current of 0.5–2 mA is delivered for around 20 min. Depending upon the parameters of the stimulation, rTMS and tDCS can increase or decrease cortical excitability and can cause neuroplastic effects. While low-frequency rTMS inhibits cortical neuronal activity, high-frequency rTMS facilitates cortical excitability. Similarly, anodal tDCS increases neuronal excitability by reducing the resting membrane threshold of cortical neurons, while cathodal tDCS decreases neuronal excitability. In contrast, tACS delivers a rhythmic current flow that can entrain pathological brain oscillations (Ingrid et al., 2014; Teo et al., 2017). In tACS, biphasic sinusoidal alternating current is used. However, in tPCS, unidirectional monophasic (although it can be bidirectional/biphasic) rectangular pulses of current are delivered. Thus, in tPCS, the stimulation is interrupted at regular intervals, defining the pulse duration, frequency, and inter-pulse intervals (IPI) of stimulation (Fitzgerald, 2014). Finally, tRNS uses an alternate current of random and constantly changing amplitude and frequency (Ingrid et al., 2014) (Figure 1).

This review highlights the application of tES specifically in movement disorders (Table 1). As the literature on tES is segregated, a heterogenous patient population has been studied, and diverse protocols have been followed (Table 2), it is difficult to write a systemic review. Despite that, we have searched for peer-reviewed articles using PubMed, BioMed Central, Cochrane Library, and ScienceDirect databases to consolidate the literature on the use of different modes of tES in the field of movement disorders. We have explained their electrophysiological basis and also highlighted the unmet needs for promoting tES as a new therapeutic intervention.

**TABLE 1:** Summary on the use of transcranial electrical stimulation (tES) in movement disorders.

Mode of tES	Used in
Transcranial direct current stimulation	1. Parkinson’s disease • Gait and balance (Benninger et al., 2010; Verheyden et al., 2013; Capecci et al., 2014; Kaski et al., 2014a; Mak and Yu, 2014; Manenti et al., 2014; Valentino et al., 2014; Costa-Ribeiro et al., 2016, 2017; Schabrun et al., 2016; Swank et al., 2016; Fernandez-Lago et al., 2017; Lattari et al., 2017; Criminger et al., 2018; da Silva et al., 2018; Dagan et al., 2018; Harris et al., 2018; Yotnuengnit et al., 2018; Alizad et al., 2019; Putzolu et al., 2019) • Upper limb function (Fregni et al., 2006; Benninger et al., 2010; Doruk et al., 2014; Salimpour et al., 2015; Costa-Ribeiro et al., 2016; Ferrucci et al., 2016; Schabrun et al., 2016; Cosentino et al., 2017; Ishikuro et al., 2018; Broeder et al., 2019) • Cognition (Nitsche et al., 2005; Boggio et al., 2006; Biundo et al., 2015; Manenti et al., 2016; Elder et al., 2017; Lawrence et al., 2018; Adenzato et al., 2019) • Impulsive pathological gambling behavior (Benussi et al., 2017a) • Speech (Pereira et al., 2013) • Sleep (Hadoush et al., 2018) • Fatigue (Forogh et al., 2017) • Dyskinesia (Kishore and Popa, 2014) 2. Multisystem atrophy-Parkinsonian type (motor disability and bradykinesia) (Alexoudi et al., 2018) 3. Corticobasal syndrome (language) (Manenti et al., 2015) 4. Progressive supranuclear palsy (language) (Madden et al., 2019; Valero-Cabré et al., 2019; Cotelli et al., 2020; de Aguiar et al., 2020) 5. Lewy body dementia (Elder et al., 2016, 2019) 6. Focal hand dystonia (Quartarone et al., 2005; Buttkus et al., 2010, 2011; Benninger et al., 2011; Furuya et al., 2014; Sadnicka et al., 2014; Bradnam et al., 2015; Rosset-Llobet et al., 2015; Marceglia et al., 2017) 7. Cervical dystonia (McCambridge and Bradnam, 2018; Summers et al., 2018) 8. Cerebellar ataxia (Grimaldi and Manto, 2013; Grimaldi et al., 2014b; Benussi et al., 2015, 2017b, 2018; Bodranghien et al., 2017; Hulst et al., 2017; John et al., 2017; Maas et al., 2019; Pilloni et al., 2019; Vavla et al., 2019) 9. Essential tremor (Gironell et al., 2014; Helvaci et al., 2016) 10. Orthostatic tremor (Lamy et al., 2018) 11. Huntington’s disease (cognitive dysfunction) (Eddy et al., 2017)
Transcranial alternating current stimulation	1. Parkinson’s disease (motor and cognitive) (Brittain et al., 2013; Krause et al., 2013; Del Felice et al., 2019)
	2. Enhanced physiological tremor (Mehta et al., 2014; Ahmad et al., 2018)
	3. Cervical dystonia (Zaghi et al., 2010; Angelakis et al., 2013)
Transcranial pulsed current stimulation	Parkinson’s disease (gait and balance) (Alon et al., 2012)
Transcranial random noise stimulation	Parkinson’s disease (cognition) and multisystem atrophy-Parkinsonian type (autonomic dysfunction) (Yamamoto et al., 2005)

**TABLE 2:** Summary on the studies of transcranial electrical stimulation in movement disorders along with the protocol used and the proposed electrophysiological basis of each of them.

Stimulation method	Proposed mechanism	Tested in	Beneficial effects seen with the protocol
Transcranial direct current stimulation	• Anodal transcranial direct current stimulation: 1. reduces the resting membrane threshold of cortical neurons, resulting in an increase in neuronal excitability 3. may induce dopamine release in the basal ganglia by activation of glutamatergic corticostriatal fibers • Cathodal transcranial direct current stimulation decreases the neuronal excitability.	1. Parkinson’s disease • Gait and balance (Benninger et al., 2010; Verheyden et al., 2013; Capecci et al., 2014; Kaski et al., 2014a; Mak and Yu, 2014; Manenti et al., 2014; Valentino et al., 2014; Costa-Ribeiro et al., 2016, 2017; Schabrun et al., 2016; Swank et al., 2016; Fernandez-Lago et al., 2017; Lattari et al., 2017; Criminger et al., 2018; da Silva et al., 2018; Dagan et al., 2018; Harris et al., 2018; Yotnuengnit et al., 2018; Alizad et al., 2019; Putzolu et al., 2019) • Upper limb function (Fregni et al., 2006; Benninger et al., 2010; Doruk et al., 2014; Salimpour et al., 2015; Costa-Ribeiro et al., 2016; Ferrucci et al., 2016; Schabrun et al., 2016; Cosentino et al., 2017; Ishikuro et al., 2018; Broeder et al., 2019) • Cognition (Nitsche et al., 2005; Boggio et al., 2006; Biundo et al., 2015; Manenti et al., 2016; Elder et al., 2017; Lawrence et al., 2018; Adenzato et al., 2019) • Impulsive pathological gambling behavior (Benussi et al., 2017a) • Speech (Pereira et al., 2013) • Sleep (Hadoush et al., 2018) • Fatigue (Forogh et al., 2017) • Dyskinesia (Kishore and Popa, 2014) 2. Multisystem atrophy-Parkinsonian type (motor disability and bradykinesia) (Alexoudi et al., 2018) 3. Corticobasal syndrome (language) (Manenti et al., 2015) 4. Progressive supranuclear palsy (language) (Madden et al., 2019; Valero-Cabré et al., 2019; Cotelli et al., 2020; de Aguiar et al., 2020) 5. Lewy body dementia (Elder et al., 2016, 2019) 6. Focal hand dystonia (Quartarone et al., 2005; Buttkus et al., 2010, 2011; Benninger et al., 2011; Furuya et al., 2014; Sadnicka et al., 2014; Bradnam et al., 2015; Rosset-Llobet et al., 2015; Marceglia et al., 2017) 7. Cervical dystonia (McCambridge and Bradnam, 2018; Summers et al., 2018) 8. Cerebellar ataxia (Grimaldi and Manto, 2013; Grimaldi et al., 2014b; Benussi et al., 2015, 2017b, 2018; Bodranghien et al., 2017; Hulst et al., 2017; John et al., 2017; Maas et al., 2019; Pilloni et al., 2019; Vavla et al., 2019) 9. Essential tremor (Gironell et al., 2014; Helvaci et al., 2016) 10. Orthostatic tremor (Lamy et al., 2018) 11. Huntington’s disease (cognitive dysfunction) (Eddy et al., 2017)	• Anodal current of 1–2 mA for 7–20 min/session for two to five sessions over M1/left dorsolateral prefrontal cortex/combined, multitargeting; some with physiotherapy • Anodal transcranial direct current stimulation to M1/bilateral M1/premotor cortex or prefrontal cortex 1–2 mA current for 20–25 min, single/multiple sessions • Anodal current of 1–2 mA for 20–25 min/session for 10–16 sessions over the left dorsolateral prefrontal cortex • Cathodal transcranial direct current stimulation over the right dorsolateral prefrontal cortex (single session, 2 mA for 10 min starting 2 min before and covering all of the task, current density 0.057 mA/cm^2^) • Increased connectivity in verbal fluency network by anodal transcranial direct current stimulation to the left dorsolateral prefrontal cortex (2 mA, 20 min) • Bilateral anodal transcranial direct current stimulation simultaneously over the left and the right prefrontal and motor areas (10 sessions, 20 min each, five per week) • Left anodal and right cathodal current of 0.06 mA/cm^2^ to dorsolateral prefrontal cortex for 20 min/session for eight sessions • Anodal current of 2 mA to cerebellum and M1 for 20 min/session for five sessions • Anodal current of 2 mA to M1 and premotor cortex for 30 min/session for 10 sessions • Anodal current of 2 mA to the left parietal cortex for 7 min, single session • Anodal current of 1.5 mA to the left dorsolateral prefrontal cortex or cathodal current to the right dorsolateral prefrontal cortex for 20 min for one to four sessions • Improvement in attentional tasks noted with anodal transcranial direct current stimulation over the left dorsolateral prefrontal cortex (single 20-min session) • Cathodal current to bilateral M1-premotor cortex/anodal current to cerebellum/cathodal current to affected motor cortex and anodal to unaffected/cathodal current to left parietal cortex and anodal current to right parietal cortex; 2 mA current for 20–24 min/session for 5 to 10 sessions • Insufficient data; ongoing studies with anodal current to the cerebellum • Anodal current of 1–2 mA to the cerebellum/cerebellum and motor cortex for 20 min, single or multiple sessions; anodal cerebellar and cathodal spinal stimulation for 2 weeks; some combined with physiotherapy • Anodal current of 2 mA to dorsolateral prefrontal cortex and cathodal current to cerebellum for 20 min/session for 15 sessions • Single session of anodal trans-spinal transcranial direct current stimulation (at the 11th thoracic vertebra level, 2.5 mA, 20 min) • Single session of anodal transcranial direct current stimulation (1.5 mA, 15 min) over the left dorsolateral prefrontal cortex combined with cognitive training
Transcranial alternating current stimulation	Interact with or even entrain spontaneous brain oscillations in a frequency-specific manner	1. Parkinson’s disease (Brittain et al., 2013; Krause et al., 2013; Del Felice et al., 2019) 2. Enhanced physiological tremor (Mehta et al., 2014; Ahmad et al., 2018) 3. Cervical dystonia (Zaghi et al., 2010; Angelakis et al., 2013)	• Phase-locked stimulation/in 10–20 Hz frequency/according to the higher power spectra band in the electroencephalograph/stimulation for 15 min over M1/chronic stimulation for 2 weeks • At peak tremor frequency over M1 • Cathodal transcranial direct current stimulation (1.5 mA, 15 min, five sessions) followed by transcranial alternating current stimulation (two sessions of 15 min stimulation with 1.5 mA at 5–15 Hz and subsequently five daily sessions of 20 min each with 1.5 mA at 15 Hz)
Transcranial pulsed current stimulation	• Tonic effect due to induced net direct current component. Neuronal excitability is modified by tonic depolarization of the resting membrane potential. • Phasic effects by the on/off nature of pulsatile currents	Parkinson’s disease (gait and balance) (Alon et al., 2012)	• Anodal current over M1 with monophasic (unidirectional) waveform with pulse duration of 33.3 μs and an interpulse interval of 33.3 μs
Transcranial random noise stimulation	• Boosting synaptic signals, stochastic resonance, inducing long-term potentiation *via* modifying *N*-methyl-D-aspartate receptor efficacy, activation of sodium channels, neuroplasticity effects • Can possibly interfere with ongoing oscillations	Parkinson’s disease (cognition) and multisystem atrophy-Parkinsonian type (autonomic dysfunction) (Yamamoto et al., 2005)	• Noisy current alternating in duration and frequency over M1 (for Parkinson’s disease), over mastoid (galvanic stimulation for autonomic dysfunction in multisystem atrophy-Parkinsonian type)

## Transcranial Direct Current Stimulation

### Idiopathic Parkinson’s Disease

#### (a) Effect on Gait and Balance

Studies have demonstrated the altered excitability of primary motor cortex (M1) in idiopathic PD patients. The low dopaminergic state of the basal ganglia may facilitate an adaptive beneficial increase of cortical excitability to compensate for the underactive pallido-thalamo-cortical drive. Enhancing the cortical excitability by tDCS may further increase this compensatory mechanism and improve motor function. Anodal tDCS may also induce dopamine release in the basal ganglia by the activation of glutamatergic corticostriatal fibers (Siebner et al., 1999; Fregni et al., 2006; Valentino et al., 2014). Studies have been done by targeting either the motor cortex (M1, 1–2 mA, 13–30 min) (Verheyden et al., 2013; Kaski et al., 2014b; Mak and Yu, 2014; Valentino et al., 2014; Costa-Ribeiro et al., 2016, 2017; Schabrun et al., 2016; Fernandez-Lago et al., 2017; da Silva et al., 2018; Yotnuengnit et al., 2018) or the dorsolateral prefrontal cortex (DLPFC, 2 mA, 7–20 min) (Manenti et al., 2014; Lattari et al., 2017). At post-stimulation, a short-term benefit in gait was noted in most of these studies.

The role of fronto-striatal circuits has been studied in freezing of gait (FOG) in PD (Lewis and Barker, 2009). The motor, the cognitive, and the limbic circuits all converge in common output nuclei globus pallidus interna/substantia nigra pars reticulata to disinhibit pedunculopontine nucleus for locomotion. In PD, there is impaired motor and cognitive processing in the cortico-striatal parallel circuits and the intra-striatal integrative circuits (Helmich et al., 2010; Alamos et al., 2019). During walking, patients with PD are more dependent on the DLPFC for cognitive control to compensate their deficit in locomotion, and more challenging walking like dual tasking needs more DLPFC activation (Maidan et al., 2016; Stuart et al., 2018). So, targeting multiple brain regions, like the prefrontal cortex along with the motor cortex, may provide better results for the gait of PD patients (Lee et al., 2019). Multitarget tDCS may reduce decoupling between the motor network and the cognitive network, improve connectivity between the prefrontal cortex, the motor cortex, and the subcortical structures and may also increase extra-striatal dopamine release (Dagan et al., 2018). Studies with multitargeting have been done with tDCS to the bilateral premotor cortex (PMC) and M1 (1 mA, 20 min) (Alizad et al., 2019), the bilateral PMC and M1 or the prefrontal cortex (PFC) separately (2 mA, 20 min) (Benninger et al., 2010), the bilateral DLPFC (2 mA, 20 min) (Swank et al., 2016; Criminger et al., 2018), the bilateral PFC separately (2 mA, 20 min) (Capecci et al., 2014), and M1 and the left DLPFC (20 min) (Dagan et al., 2018).

Postural imbalance and falls impair the quality of life of PD patients and increase the overall health cost burden. Anodal tDCS to the left DLPFC (2 mA, 20 min) has been noted to improve balance and functional mobility. The increased excitability of DLPFC may enhance visuo-spatial processing, which is responsible for improved balance and functional mobility (Lattari et al., 2017). The beneficial effect of DLPFC tDCS (2 mA, 7 min; two sessions—left and right DLPFC) in timed up and go task has also been observed in another study (Manenti et al., 2014). A single session of anodal tDCS (1.5 mA for 20 min) over the left DLPFC improved step length, stride velocity, and double support time during obstacle negotiation task (Putzolu et al., 2019).

With anodal tDCS to M1 (2 mA, 20 min) (Schabrun et al., 2016) or to bilateral DLPFC tDCS (left anodal, right cathodal; 2 mA, 20 min) (Swank et al., 2016), no significant benefit was noted in dual-task gait in PD, but tDCS has been shown to influence task prioritization on dual-task walking (Criminger et al., 2018). Anodal tDCS over the supplementary motor area (SMA, 2 mA, 13 min) prolonged the effects of cued gait training on functional mobility independent of dopaminergic medication state (Costa-Ribeiro et al., 2016).

A beneficial effect of combining tDCS (over primary motor and PMC, 2 mA, 15 min) with physical training has been demonstrated on gait velocity and balance. tDCS can lower the threshold of physical training to normalize cortical excitability in M1 (Valentino et al., 2014). A single case study showed the benefit of primary and PMC anodal tDCS on trunk peak velocity and average trunk sway during tango dancing (Kaski et al., 2014a). Combined sessions of exercise-based video gaming (exergaming) and anodal tDCS over M1 (2 mA, 20 min/session, two sessions/week for 12 weeks) improved the static and the dynamic balance in PD patients. Exercise combined with tDCS may help in motor learning and consolidation of long-term motor skill retention in PD patients (Harris et al., 2018).

#### (b) Effect on Upper Limb Motor Function

In PD, there is progressive involvement of upper limb function, mostly asymmetric to start with, manifested by impaired dexterity, abnormal force generation, and poor bimanual coordination (Ingvarsson et al., 1997; Ponsen et al., 2006; Vanbellingen et al., 2011). An improvement was noted in upper limb motor sequencing and finger tapping after anodal/cathodal tDCS over the more affected/less affected M1 (2 mA, 20 min), respectively, by Cosentino et al. (2017). On the contrary, Salimpour et al. (2015) noted an increase in force assignment to the more affected hand after bilateral tDCS with the cathode over the more affected hand and with the anode over the less affected M1 (2 mA, 25 min). A significant improvement in upper limb motor sequencing was also noted by Benninger et al. (2010) in multi-session anodal tDCS (8 sessions, 2 mA, 20 min) to bilateral M1/PMC or PFC that lasted during 3 months of follow-up. Stimulation to SMA (2 mA, 13 min) (Costa-Ribeiro et al., 2016) or DLPFC (10 sessions, 2 mA, 20 min) (Doruk et al., 2014) revealed no significant benefit.

Broeder et al. (2019) noted an increase in writing amplitude with anodal tDCS to M1 (1 mA, 20 min). Fregni et al. (2006) also noted some benefit in Purdue pegboard task with anodal tDCS to M1 (1 mA, 20 min), but other studies found no significant benefit in dexterity tasks with single- or multi-session tDCS to the fronto-polar area (five sessions, 1 mA, 15 min) (Ishikuro et al., 2018) or DLPFC (10 sessions, 2 mA, 20 min) (Doruk et al., 2014).

Upper limb reaction time improved with anodal tDCS to M1 (1 mA, 20 min) in the study by Fregni et al. (2006), but there was no significant change in reaction time noted in other studies with tDCS to M1 (nine sessions, 2 mA, 20 min) (Schabrun et al., 2016), bilateral M1 and PMC or bilateral PFC (eight sessions, 2 mA, 20 min) (Benninger et al., 2010), DLPFC (10 sessions, 2 mA, 20 min) (Doruk et al., 2014), and bilateral cerebellum (five sessions, 2 mA, 20 min) (Ferrucci et al., 2016).

Thus, the most improvement with M1 tDCS has been noted in simple motor tasks, but in complex task processing, tDCS may not be that beneficial. In complex tasks, where more cognitive load is there along with motor manipulation, stimulating DLPFC along with M1 is more rational, but for a long-term effect, multisession tDCS is needed.

#### (c) Effect on Cognition

Cognitive dysfunction is one of the most common non-motor symptoms in PD patients. Dysfunction in frontostriatal circuitry involving DLPFC and dorsal caudate, due to dopaminergic depletion, is mainly responsible for the executive dysfunction in PD (de la Fuente-Fernández, 2012). tDCS to the left dorsolateral prefrontal cortex (LDLPFC) has shown an improvement in working memory performance by enhancing the local cortical excitability. A beneficial effect of anodal tDCS has been noted in both, during the task (online effect, 2 mA, 20 min, to the left DLPFC) (Boggio et al., 2006) and after the task (offline effect, 2mA, 20 min to left/right DLPFC) (Doruk et al., 2014). While the online effects of tDCS are due to changes in polarization in neural membranes, the offline effects are related to long-term potentiation, long-term depression, and thus long-term synaptic plasticity (Nitsche et al., 2005).

In patients with Parkinson’s disease dementia (PDD), a single session of anodal tDCS to the left DLPFC (current density of 0.08 mA/cm^2^ for 20 min) failed to show any benefit in attentional tasks. Multiple stimulation sessions are likely needed to modify complex attentional network in PDD (Elder et al., 2017).

A beneficial effect of tDCS to LDLPFC (1.5–2 mA, 20 min/session, 1–4 days/week for 4 weeks) has been noted in PD with mild cognitive impairment (PD-MCI), with concurrent cognitive training (Biundo et al., 2015; Lawrence et al., 2018) and physiotherapy (anodal tDCS, 2 mA for 25 min/session, five sessions/week for 2 weeks) (Manenti et al., 2016). In PD-MCI, tDCS over the medial frontal cortex (anode over Fpz, cathode between inion and oz, 1.5 mA for 6 min, current density 0.043 mA/cm^2^) enhanced the Theory of Mind, i.e., the ability to understand and predict other people’s behaviors as assessed by the Attribution of Intentions task (Adenzato et al., 2019).

#### (d) Effect on Impulsive Pathological Gambling Behavior

Pathological gambling is one of the major side effects of dopamine agonist therapy in PD patients. The dysfunction of the orbitofrontal-ventrostriatal circuitry is likely responsible for such a risky and impulsive behavior (Gatto and Aldinio, 2019). An improvement in decision making was noted in Iowa Gambling Task with cathodal tDCS over the right DLPFC (single session, 2 mA for 10 min, starting 2 min before and covering all of the tasks, current density 0.057 mA/cm^2^). Cathodal tDCS over the right DLPFC likely reduced the pathological overdrive in frontostriatal circuitry (Benussi et al., 2017a).

#### (e) Effect on Speech

Parkinson’s disease is associated with deficits in phonemic and semantic fluency due to frontal and temporal lobar dysfunction. Modulation of verbal and phonemic fluency was noted by anodal tDCS to the left DLPFC (2 mA, 20 min) compared to the left temporo-parietal cortex. DLPFC tDCS increased the functional connectivity in verbal fluency networks involving frontal, parietal, and fusiform areas (Pereira et al., 2013).

#### (f) Effect on Sleep, Fatigue, and Daytime Sleepiness

Sleep disturbance is a common non-motor symptom in patients with PD, adversely affecting their overall quality of life and promoting neuropsychiatric complications like depression (Kay et al., 2018). Bilateral anodal tDCS simultaneously over the left and the right prefrontal and motor areas (10 sessions, 20 min each, five sessions per week) improved their Pittsburgh Sleep Quality Index total score, sleep latency sub-score, Geriatric Depression Scale total score, and physical and mental component scores of the health-related quality-of-life questionnaire (SF-36) (Hadoush et al., 2018).

A beneficial effect of bilateral DLPFC tDCS (eight sessions, 0.06 mA/cm^2^ current, 20 min/session) on fatigue in PD patients, using left anodal and right cathodal stimulation, has been shown by Forogh et al. (2017) when combined with occupational therapy. No significant effect was seen on daytime sleepiness. The effect on fatigue could have been due to an improvement in mood and depressive symptoms.

#### (g) Effect on Dyskinesia

An involvement of the cerebello-thalamo-cortical circuit and its aberrant plasticity has been noted in L-dopa-induced dyskinesia (LID) (Yoo et al., 2019). An improvement in LID has been shown with a combined effect of increased cerebellar inhibition (CBI) by cerebellar anodal tDCS (2 mA for 20 min/day for five consecutive days) and modulation of motor cortical excitability by M1 tDCS (Ferrucci et al., 2016). Cerebellar stimulation may help in restoring the cerebellar and the basal ganglionic control over the non-salient inputs to the motor areas during pulsatile dopaminergic surges (Kishore and Popa, 2014).

So, there is a huge scope of exploring tDCS in PD. Even with the best use of dopaminergic drugs, symptoms like FOG, non-motor symptoms, and drug-induced dyskinesia are difficult to manage. tDCS is affordable, portable, cost-effective, and well tolerated. The unmet need is to have a clearer idea of the site of stimulation (M1/DLPFC/cerebellum) and the necessary parameters of stimulation (intensity, duration) from rigorous blinded studies in larger datasets.

### Parkinson’s Plus Syndromes

Parkinson’s plus syndromes unlike idiopathic PD do not respond well to levodopa. Evaluating a new therapeutic intervention, such as NIBS, is worthwhile to provide some symptomatic benefit in these disabling disorders.

A lasting beneficial effect on walking speed and leg bradykinesia was noted with anodal tDCS to the motor and the pre-motor cortex (2 mA, 30 min, in 10 sessions over 2 weeks) in a patient with multisystem atrophy-Parkinsonian type (MSA-P) (Alexoudi et al., 2018).

The effect of anodal tDCS over the bilateral parietal cortex on naming performance was evaluated in corticobasal syndrome (CBS) with linguistic deficits. A shortening of action naming latency was observed only after anodal stimulation over the left parietal cortex (Manenti et al., 2015).

Anodal tDCS over the left DLPFC (in four sessions) was used to improve non-fluent aphasia in the case of progressive supranuclear palsy (PSP). An improved performance was seen in phonemic fluency, action naming, and speech production (Madden et al., 2019). Recently, tDCS has been further explored on language processing in PSP by its ability to modulate prefrontal brain networks. Valero-Cabré et al. (2019) has shown a short-term improvement of semantic (category judgment) and lexical (letter fluency) skills by a single session of right cathodal tDCS and left anodal tDCS to the DLPFC, respectively. A combined effect of tDCS and language training has improved naming performance in primary progressive aphasia (PPA) (Cotelli et al., 2020). In PPA, the brain volumes of specific anatomic areas influenced the beneficial effect of anodal tDCS over the left inferior frontal gyrus when combined with written naming/spelling therapy (de Aguiar et al., 2020).

In Lewy body dementia (LBD), an improvement in attentional tasks (choice reaction time and digit vigilance) was noted with anodal tDCS over the left DLPFC (single 20-min session, 0.08 mA/cm^2^), but not in visuoperceptual task performance (Elder et al., 2016). Repeated consecutive sessions (two consecutive 20-min sessions, 0.048 mA/cm^2^, for 5 days) of parietal anodal tDCS and occipital cathodal tDCS failed to improve visual hallucination, visuoperceptual function, or occipital cortex excitability in LBD (Elder et al., 2019).

Presently, PD plus syndromes are difficult to manage. Neither dopaminergic drugs nor deep brain stimulation (DBS) is a useful option. Data using non-invasive stimulation methods for treatment are minimal. Collaborative studies using standardized protocols are now needed to explore these options.

### Dystonia

The excessive and inappropriate muscle activation patterns in dystonia reflect the disinhibition of cortical–subcortical motor circuits, which is a consequence of abnormal sensorimotor integration and maladaptive plasticity (Hallett, 2006). A deficiency in short intracortical inhibition, likely a GABAergic effect, has been demonstrated in some studies, with paired-pulse stimulation (Hallett, 2011). Thus, the down-regulation of cortical excitability by cathodal tDCS seems to be a good therapeutic option in dystonia.

Beneficial effects were noted with bilateral cathodal tDCS (2 mA, 20 min/day for five consecutive days) over the motor–premotor cortex in musicians with focal hand dystonia. Bilateral cathodal tDCS could have downregulated the cortical excitability responsible for excessive excitation and near-synchronous co-contractions of agonists and antagonists (Marceglia et al., 2017).

The cerebellum has been another target for modulation in dystonia due to its inhibitory effect over the motor cortex. Anodal tDCS over the cerebellar hemispheres (2 mA, 20 min) has been shown to reduce the average pen pressure and modify the mean stroke frequency during handwriting and fast cyclic drawing in patients with focal hand dystonia (Bradnam et al., 2015). There are also some studies of cerebellar anodal tDCS in cervical dystonia (CD), but with insufficient data for therapeutic use (McCambridge and Bradnam, 2018; Summers et al., 2018).

On the contrary, some studies have shown no benefit of tDCS in dystonia. The permanent maladaptive plasticity in chronic dystonia may be responsible for the irreversible changes and loss of efficacy of tDCS (Quartarone et al., 2005).

A study by Benninger et al. (2011) of the contralateral primary motor cortex cathodal stimulation (2 mA, 20 min, three sessions within 1 week) in writer’s cramp patients failed to show any improvement. When anodal cerebellar tDCS (ctDCS; 2 mA, 15 min) was tried simultaneously with paired associative stimulation *via* transcranial magnetic stimulation (TMS) in patients with writing dystonia/writers’ cramp, the clinical symptoms were unchanged (Sadnicka et al., 2014).

Combining tDCS with neurorehabilitation is another approach. tDCS may prime the central somatosensory pathways, promote their plasticity, and facilitate surrounding inhibition in the hyperactive areas, rendering them more responsive to neurorehabilitation.

When tDCS to bilateral motor cortices (cathodal to affected cortex, anodal to unaffected cortex; 2 mA, 24 min) was tried in pianists with focal dystonia, improvement was seen in the rhythmic accuracy of sequential finger movements only when concurrent motor training was done. Fine motor control of the hand affected by focal dystonia can thus be improved by (i) facilitation of the transcallosal inhibitory input into the affected cortex by activating the unaffected motor cortex by anodal stimulation and (ii) suppression of abnormal hyperactivity in the affected motor cortex by cathodal stimulation (Furuya et al., 2014).

In a study on musician’s focal dystonia, retraining with slow, voluntarily controlled movements on the piano was combined with tDCS (2 mA, 20 min) to contralateral primary motor cortex (C3). No beneficial effect was noted with single-session tDCS. However, a single retraining session of 20 min with tDCS may not be sufficient to modify the sensorimotor learning of a highly skilled task like in musician’s dystonia (Buttkus et al., 2011). In another similar study by the same group, some improvement was seen in one of the 10 patients with atypical arm dystonia rather than focal hand dystonia (Buttkus et al., 2010).

A significant improvement in dystonia severity score was noted with a 2-week combined therapy of neurorehabilitation by sensory motor retuning (SMR) and biparietal tDCS (cathode over the left and anode over the right; 2 mA for 20 min/session for the first 30 min of the 1-h daily SMR session) (Rosset-Llobet et al., 2015).

In summary, tDCS by itself or in combination with rehabilitation therapy can be an effective way to modulate the dysfunctional network of dystonia. Cathodal stimulation seems more rational. Parameters like site of stimulation (M1/SMA/cerebellum), duration, and sustainability of stimulation are to be evaluated in further research. Unlike Parkinsonian syndromes, dystonia can be focal or generalized. It is likely that the site of stimulation and the effects would be substantially different in focal *versus* generalized dystonia. A careful mapping of these abnormalities to differentiate the syndromes physiologically would be required.

### Cerebellar Ataxia

Cerebellar tDCS can modulate the excitability of Purkinje cells in the cerebellar cortex and hence modify the cerebellar output *via* the cerebello-thalamo-cortical pathway. A polarity-specific effect has been shown in different studies. Anodal ctDCS increases the excitability of Purkinje cells of the cerebellar cortex, augmenting the inhibitory effects of the cerebellar cortex on the deep cerebellar nuclei and, hence, reducing the cerebello-thalamic facilitatory drive to the cortical areas (Grimaldi et al., 2014a, 2016; Maas et al., 2020).

An initial study by Grimaldi and Manto (2013) failed to show any significant effect of cerebellar anodal tDCS on upper limb coordination and posture. However, anodal ctDCS (1 mA, 20 min) reduced the amplitudes of long-latency stretch reflexes. Subsequently, in a separate study by the same group, a beneficial effect of the cerebello-cerebral tDCS (tCCDCS; 1 mA, 20 min on each site) has been demonstrated on upper limb tremor (postural and action) by power spectral density analysis. tCCDCS reduced the onset latency of the antagonist activity associated with fast goal-directed movements toward three aimed targets (Grimaldi et al., 2014b). Hence, tCCDCS modified the delayed-onset braking action of antagonist activity that results in hypermetria in cerebellar ataxia.

A transient beneficial effect of single-session ctDCS (2 mA, 20 min) on degenerative cerebellar ataxia has been demonstrated by Benussi et al. (2015). Subsequently, in a different crossover study by the same group, they have demonstrated long-term effects of anodal ctDCS using stimulation for 5 days/week for 2 weeks (Benussi et al., 2017b). Recently, they have also used cerebello-spinal tDCS with anodal cerebellar and cathodal spinal stimulation. CBI was measured using TMS. Statistically significant beneficial effects were seen in short term (2 weeks) and long term (3 months). The benefit is likely due to the combined effect of CBI by anodal ctDCS and influence on the ascending and the descending spinal pathways on spinal reflex excitability and functional neuroplastic changes by spinal cathodal tDCS (Benussi et al., 2018). The results of a similar trial with 2 weeks of anodal ctDCS in patients with spinocerebellar ataxia type 3 are awaited (Maas et al., 2019). An improvement of marked postural tremor in cerebellar ataxia associated with ANO10 mutation was noted with cerebello-cerebral stimulation (anode over cerebellum and cathode over M1, 1.5 Ma, 20 min) (Bodranghien et al., 2017). No significant beneficial effect was noted with anodal tDCS to the cerebellum or the motor cortex in grip force control (2 mA, 25 min) (John et al., 2017) or force-field reaching adaptation (2 mA, 22 min) (Hulst et al., 2017) in patients with cerebellar ataxia.

A beneficial effect of combined intensive rehabilitation program (IRP) and cerebello-cerebral tDCS was seen in patients with Friedreich’s ataxia. IRP consisted of two sessions/day for 5 weeks, and tDCS (2 mA, 20 min) was applied once/day for 2 weeks. tDCS can facilitate the rehabilitative interventions, likely by improving the recruitment activity at the pyramidal cell layer on the M1 with subsequent neural network function recovery (Vavla et al., 2019). A similar positive effect was noted in home-based chronic stimulation with remotely supervised anodal ctDCS (2.5 mA, 20 min, 60 sessions) with cognitive and physiotherapy in an elderly female with progressive cerebellar ataxia (Pilloni et al., 2019).

We believe that there is a huge scope of using tDCS in degenerative cerebellar ataxia where practically no therapeutic options are available. Isolated cerebellar stimulation, or in combination (cerebello-cerebral and cerebello-spinal), can be offered in ataxic patients. A concurrent rehabilitation program may boost the therapeutic benefit.

### Essential Tremor

There are two circuits implicated in tremor generation: (de Schipper et al., 2018) the cortico-ponto-cerebello-thalamo-cortical loop and (Schirinzi et al., 2018) the Guillain–Mollaret triangle circuit. The two circuits interact in the cerebellum (Dietrich and Hallett, 2018). In essential tremor (ET) patients, decreased functional connectivity was noted between the cerebellar cortex and the dentate nucleus, with an increase of functional connectivity between the cerebellar cortex and the thalamus (Buijink et al., 2015; Gallea et al., 2015; Maas et al., 2020).

In ET patients, by anodal tDCS to dorsolateral prefrontal areas (Cz and F4) and cathodal stimulation to inion (2 mA, 20 min/session, total of 15 sessions), a significant improvement is seen in the Essential Tremor Rating Assessment Scale and the Activities of Daily Living scores (Helvaci et al., 2016), although in another study in ET patients, using cathodal tDCS to both cerebellar hemispheres and anode over both prefrontal areas (2 mA, 20 min/session, 10 sessions), no significant improvement occurred (Gironell et al., 2014).

The effects of tDCS on tremor may not be noticed acutely and may require a long follow-up period to appreciate. Further studies with larger sample sizes to evaluate the efficacy of tDCS in ET in the short and the long term are needed.

### Orthostatic Tremor

Classic > 13 Hz orthostatic tremor (OT) in the legs is believed to be due to a deficit in proprioceptive feedback to the sensory–motor cortex, where medications are of limited value. DBS to caudal zona incerta and spinal cord stimulation are newer options targeting that defective sensory feedback (Krauss et al., 2006; Gilmore et al., 2019). Lamy et al. (2018) noted an improvement in the amplitude of tremor and instability in OT patients with a single session of anodal trans-spinal tDCS (2.5 mA, 20 min).

### Huntington’s Disease

Cognitive impairment, especially deficit in working memory, may precede motor impairment in Huntington’s disease (HD). A reduced activation of the left DLPFC has been noted in HD (Georgiou-Karistianis et al., 2013). A single session of anodal tDCS (1.5 mA, 15 min) over the left DLPFC combined with cognitive training improved the patients’ performance on a working memory task (digit reordering task) (Eddy et al., 2017).

Abnormal cortical excitability and plasticity have been demonstrated in the early phase of HD and in asymptomatic HD carriers. A study with low-frequency (1 Hz) rTMS to the SMA improved chorea in HD (Brusa et al., 2005). Like in L-dopa induced dyskinesia, modulation of cortical excitability with tDCS can be tried in HD chorea.

Overall, the transcranial delivery of anodal tDCS can excite the underlying hypoactive brain region, and cathodal tDCS can suppress the hyperactivity. So, the efficacy of tDCS depends on the right choice of the polarity of tDCS based on the hypo- or hyper-activity of the underlying brain network.

## Transcranial Alternating Current Stimulation

### Parkinson’s Disease

Studies using electrophysiological recordings in cortico-basal ganglia circuits have demonstrated that the loss of dopamine in PD increases the sensitivity of the basal ganglia-thalamo-cortical network to rhythmic oscillatory inputs. This possible cortically originating rhythm leads to pathological oscillatory synchronization and thus interferes with the processing of movement-related signals, resulting in motor deficits. Bradykinesia and rigidity in PD are likely related to increased oscillatory beta band synchronization (Feurra et al., 2011; Weinberger and Dostrovsky, 2011). Cross-frequency phase-amplitude coupling between amplitude of slow high frequency oscillation (200–300 Hz) and phase of low-beta (13–22 Hz) has been noted in the OFF phase of PD (López-Azcárate et al., 2010). tACS has been proposed to interact with or even entrain spontaneous brain oscillations in a frequency-dependent manner by the subthreshold modulation of membrane potentials (Teo et al., 2017).

An improvement in motor and cognitive performance in PD was noted with individualized tACS and physiotherapy, where the frequency of tACS was set according to the higher-power spectra band in the electroencephalograph (EEG; 4 Hz tACS if beta excess on EEG map; 30 Hz tACS if theta excess, 5 days/week for 2 weeks) (Del Felice et al., 2019). Brittain et al. (2013) have studied the role of tACS in suppressing rest tremor in 12 PD patients by applying a phase cancellation technique. At first, they identified the timing of cortical oscillations responsible for the rest tremor by delivering tremor-frequency stimulation over M1—the rhythms drift in and out of phase alignment with one another. tACS was delivered at these specified phase alignments to demonstrate around 50% suppression of the ongoing resting tremor amplitude.

In a different study, the effects of 10 and 20 Hz tACS over M1 (1 mA, 15 min, current density 0.0286 mA/cm^2^, sinusoidal waveform) have been evaluated on magnetoencephalographic recording during isometric contraction of the forearm muscles (corticomuscular coupling, CMC) and motor tasks (fast finger tapping and wrist pronation–supination) in PD patients. Decreased beta band CMC and variability of fast distal movements were noted with M1 tACS at 20 Hz in PD patients, possibly because of pathological beta synchronization of the motor cortex in PD (Krause et al., 2013).

Recently, cross-frequency phase-amplitude coupling between phase of theta–alpha (4–12 Hz) and amplitude of fast high frequency oscillation (300–400 Hz) has been demonstrated in the ON state of PD with dyskinesia (Ozturk et al., 2020). So, there is a scope of tACS to evaluate its role also in the ON state of dyskinesia.

### Enhanced Physiological Tremor

Mehta et al. (2015) have highlighted the importance of montage selection for entraining physiological tremor. The montage with active electrode over M1 and extracephalic reference electrode contralateral to M1 significantly entrained the physiological tremor. tACS was delivered at the participant’s peak tremor frequency. In a different study, they have evaluated the effect of tACS (delivered at the task-dependent peak frequency of tremor) on the postural and the kinetic type of physiological tremor. M1 stimulation gave rise to phase entrainment of postural, but not kinetic, tremor, whereas cerebellar stimulation increased entrainment in both cases. However, tACS had no effect on the amplitude of physiological tremor, which may be because of a dominant role of factors further downstream of the central oscillators in the modulation of tremor amplitude (Mehta et al., 2014). It has been shown that focused M1 tACS caused a significant phase entrainment of the tremor, and the subjects with higher phase entrainment showed more tremor amplitude modulation (Ahmad et al., 2018).

### Cervical Dystonia

Transcranial alternating current stimulation (tACS) over the motor cortex is noted to significantly decrease the amplitude of motor-evoked potentials and decreased intracortical facilitation. tACS (15 Hz, 20 min) may have a dampening effect on the cortical networks, and it likely interferes with the temporo-spatial summation of weak subthreshold electric potentials (Zaghi et al., 2010).

With stimulation of the motor cortex by cathodal tDCS (1.5 mA, 15 min, five sessions) followed by tACS (two sessions of 15-min stimulation with 1.5 mA at 5–15 Hz and subsequently five daily sessions of 20 min each with 1.5 mA at 15 Hz), a 54% reduction in the Toronto Western Spasmodic Torticollis Rating Scale (TWSTRS) and a 75% reduction in the TWSTRS Pain Scale was noted in a patient of idiopathic cervical dystonia, and the effects persisted at 30 days of follow-up (Angelakis et al., 2013).

Neural entrainment and plasticity are mainly suggested to mediate the effects of tACS, and there is potential scope for using it as a possible treatment for disorders related to malfunctioned brain oscillations. Frequency, intensity, and duration of stimulation are yet to be standardized.

## Transcranial Pulsed Current Stimulation

While anodal-tDCS modifies neuronal excitability by tonic depolarization of the resting membrane potential, anodal-tPCS (a-tPCS) modifies neuronal excitability by a combination of tonic and phasic effects. The tonic effects of a-tPCS are related to the net direct current component, leading to the tonic depolarization of the resting membrane potential. The phasic effects of a-tPCS are due to the on/off nature of pulsatile currents. In tPCS, the current flows in unidirectional pulses separated by an IPI, in contrast to the continuous flow of direct current in tDCS (Fitzgerald, 2014; Jaberzadeh et al., 2015). tPCS can be applied with short inter-pulse intervals (tPCSSIPI) or long inter-pulse intervals (tPCSLIPI).

Jaberzadeh et al. (2014) tested four testing conditions: a-tDCS, a-tPCSSIPI (IPI 50 ms), a-tPCSLIPI (IPI 650 ms), and sham a-tPCSSIPI. They have noted that only anodal tDCS and anodal tPCSSIPI over M1 increase the corticospinal excitability in healthy individuals, lasting for at least 30 min. The increase in CSE was larger with a-tPCSSIPI.

The effect of tPCS (with a commercially available tPCS device—Fisher Wallace model FW 100-C, New York) with treadmill walk has been evaluated in PD patients, with focus on gait and balance. The tPCS session increased gait velocity and stride length significantly compared with treadmill or tPCS + treadmill. The number of steps needed to recover balance decreased after tPCS and tPCS + treadmill (Alon et al., 2012).

The intensity-specific modulation of cortical excitability by tPCS has been addressed recently by Ma et al. (2019). Enhancement of cortical excitability by low-intensity anodal tPCS is likely related to astrocytic Ca^2+^ elevations due to the noradrenergic activation of alpha-1 adrenergic receptors, but high-intensity anodal tPCS decrease cortical excitability with excessive calcium activity in neurons.

The role of tPCS in other disorders like ataxia, where gait and balance are predominantly affected, has not been studied to date.

## Transcranial Random Noise Stimulation

The newest mode of NIBS is tRNS. tRNS over M1 has been shown to enhance corticospinal excitability both during and after stimulation in the healthy human brain. The main advantage of tRNS is the direction insensitivity of the stimulation. Boosting synaptic signals, stochastic resonance, activation of sodium channels, and inducing long-term potentiation *via* modifying *N*-methyl-D-aspartate receptor efficacy are likely responsible for the neuroplasticity effects. tRNS, like tACS, can possibly interfere with ongoing oscillations and neuronal activity in the brain (Terney et al., 2008).

Yamamoto et al. (2005) have evaluated the effect of 24-h noisy galvanic vestibular stimulation (GVS) on long-term heart rate dynamics in patients with multisystem atrophy (MSA) and on daytime trunk activity dynamics in patients with PD. They have noted improved autonomic, especially parasympathetic, responsiveness by noisy GVS. The cognitive performance in those PD patients has also been evaluated by means of a continuous performance test. The mean reaction time of the continuous performance test was significantly decreased by the noisy GVS, suggesting improved motor execution during the cognitive task.

Motor cortex plasticity in PD patients has been studied with tRNS and intermittent theta-burst stimulation (iTBS). The plasticity-inducing effect of iTBS was absent in PD patients, but tRNS reduced cortical excitability as compared to pre-stimulation baseline, which is in contrast to the results on healthy subjects (Stephani et al., 2011). Recently, Moret et al. (2019) has noted that, for inducing a significant and persistent increase in cortical excitability, a large amount of noise with a wide range of frequencies (100–700 Hz) is needed.

The altered cortical plasticity in PD patients should be evaluated further. On the other hand, in dystonia, there is definite evidence of maladaptive cortical plasticity and defective sensory–motor integration. Exploring the role of tRNS in these disorders of altered cortical plasticity will be an important next step.

## Conclusion

The results of tES studies in movement disorders are encouraging, but their utility in the mainstream treatment of movement disorders is still limited. Because of the heterogeneity of patient population and the diversity of the protocols used in these studies, it is hard to do a systemic review and to quantify the actual therapeutic benefit of different modes of tES. The majority of trials are not double-blinded and the level of evidence of efficacy and safety is unknown. These are the major limitations for reviewing different modes of tES. So far, tDCS is the most commonly used technique. In the field of movement disorders, tDCS has been tested mostly for different aspects of PD (22 studies targeting gait and balance, 10 studies evaluating upper limb motor function, seven studies for cognitive function, and one study each for pathological gambling, speech, sleep, fatigue, and dyskinesia). The efficacy of tDCS has also been tested in dystonia and cerebellar ataxia (11 studies for each), but the number of studies for other movement disorders like ET, OT, HD, MSA, PSP, and CBS is quite less. Anodal tDCS over motor cortex in PD, over cerebellum in ataxia (±simultaneous cathodal spinal stimulation) and cathodal tDCS over motor cortex in dystonia, has shown beneficial results. Modifying a complex dysfunctional network by acute stimulation seems unlikely. Chronic stimulation for at least 2 weeks seems to be a safe and rational approach. Other techniques like tACS, tPCS, and tRNS are less well studied in movement disorders. As tACS has been proposed to entrain brain oscillations, it can be used as a tool to assess and modulate the complex tremor network. Recently, cross-frequency phase-amplitude coupling is an evolving pathophysiology for the OFF and the ON state of PD, which further expands the scope of tACS in PD. So far, tACS has been evaluated in PD (three studies), enhanced physiological tremor (two studies), and cervical dystonia (two studies). In contrast, tPCS and tRNS are relatively new in the field of movement disorders. Due to its combined phasic and tonic effects, tPCS can be an effective and more tolerable therapy in PD or ataxia. The role of tRNS with a wide range of frequency should also be evaluated further in movement disorders.

Newer technologies like quantitative electroencephalography, better circuit design for stimulation devices, and programmability of stimulation parameters are all necessary to move this field forward. Elucidating the precise patterns of network dysfunction in a highly connected system is another important engineering problem that has to be directly tackled by novel signal processing methods. It may only then be possible to target the precise individualized sites in specific patients with specific diseases for stimulation. Considered together, this emerging field of individualized dysfunction measurement and device optimization for portable non-invasive stimulation for movement disorders will be the next frontier of tES.

In the literature, there are mostly segregated case reports and reviews on tES. In them, mostly tDCS has been focused, while other modes are neglected. There is no literature combining all the modes of tES together so far. In this review, we have highlighted the basic concept of the different modes of tES and have summarized the studies done so far on the therapeutic benefit of tES in movement disorders. We have also tried to find out the electro-physiological basis of the effect of each of these techniques. There still remain some unanswered questions: (de Schipper et al., 2018). What are the detailed electrophysiological bases of the different modes of tES and are they sufficient enough to alter the complex brain network of movement disorders? (Schirinzi et al., 2018). Are there any methods like quantitative EEG, ligand-bound imaging, *etc*., to probe into the network for planning individualized tES? (Benito-León et al., 2015). Is there any threshold of neurodegeneration beyond which applying tES is not reasonable? (Wu et al., 2018). What would be the realistic expectation after tES? (Falcon et al., 2016). How long does the effect of stimulation last? (Latorre et al., 2019). How feasible is supervised home-based chronic stimulation by patients themselves and is there any scope of adaptive tES as per patient need? Large clinical trials with each of the stimulation technique, precisely targeting the individualized brain network, may help us to find some of the answers and thus will help to set up a standardized protocol for each of them.

### Take-Home Message

1. Non-invasive tES may be a safe and cost-effective way to alter cortical excitability.
2. Anodal tDCS over motor cortex in PD, over cerebellum in ataxia (± simultaneous cathodal stimulation over spinal cord), and cathodal tDCS over motor cortex in dystonia have shown beneficial results.
3. While acute stimulation may cause a transient effect, for sustained benefit, chronic stimulation for at least 2 weeks may be required.
4. tACS can entrain pathological brain oscillations.
5. More trials with tPCS and tRNS are needed to evaluate their efficacy.

## Author Contributions

JG wrote the first draft of the manuscript. AM, SS, and DA contributed to the review and critique. MJ contributed to the conception of the work, review, and critique.

## Conflict of Interest

The authors declare that the research was conducted in the absence of any commercial or financial relationships that could be construed as a potential conflict of interest.
